# Short-term evaluation of immune levels and nutritional values of EN versus PN in gastric cancer: a systematic review and a meta-analysis

**DOI:** 10.1186/s12957-019-1658-9

**Published:** 2019-07-03

**Authors:** Fan Xin, Said Abdulrahman Salim Mzee, Godwin Botwe, Han He, Sun Zhiyu, Chen Gong, Said Twahir Said, Chen Jixing

**Affiliations:** 1 Affiliated Hospital of Jiangsu University, Zhenjiang, Jiangsu People’s Republic of China; 2 Jiangsu University, Zhenjiang, Jiangsu People’s Republic of China; 3 Msambweni County Referral Hospital, Kwale County, Kenya; 4 Overseas Education College, Jiangsu University, No. 301 Xuefu Road, Zhenjiang, 212013 Jiangsu People’s Republic of China

**Keywords:** EN feeding tubes, PN, ERAS, Gastrectomy, Gastric cancer

## Abstract

**Background:**

Postsurgical patients’ oral feeding begins with clear fluids 1–3 days after surgery. This might not be sufficiently nutritious to boost the host immune system and provide sufficient energy in gastric neoplastic patients to achieve the goal of enhanced recovery after surgery (ERAS). Our objective was to analyze the significance of early postoperative feeding tubes in boosting patients’ immunity and decreasing incidence of overall complications and hospital stay in gastric cancer patients’ post-gastrectomy.

**Methods:**

From January 2005 to May 24, 2019, PubMed and Cochrane databases were searched for studies involving enteral nutrition (EN) feeding tubes in comparison to parenteral nutrition (PN) in gastric cancer patients undergoing gastrectomy for gastric malignancies. Relative risk (RR), mean difference (MD), or standard mean difference (SMD) with 95% confidence interval (CI) were used to estimate the effect sizes, and heterogeneity was assessed by using *Q* and *χ*^2^ statistic with their corresponding *P* values. All the analyses were performed with Review Manager 5.3 and SPSS version 22.

**Results:**

Nine randomized trials (*n* = 1437) and 5 retrospective studies (*n* = 421) comparing EN feeding tubes and PN were deemed eligible for the pooled analyses, with a categorized time frame of PODs ≥ 7 and PODs < 7. Ratio of CD4+/CD8+ in EN feeding tubes was the only outcome of PODs < 7, which showed significance (MD 0.22, 95% CI 0.18–0.25, *P* < 0.00001). Regarding other immune indicators, significant outcomes in favor of EN feeding tubes were measured on POD ≥ 7: CD3+ (SMD 1.71; 95% CI 0.70, 2.72; *P* = 0.0009), CD4+ (MD 5.84; 95% CI 4.19, 7.50; *P* < 0.00001), CD4+/CD8+ (MD 0.28; 95% CI 0.20; 0.36, *P* < 0.00001), NK cells (SMD 0.94; 95% CI 0.54, 1.30; *P* < 0.00001), nutrition values, albumin (SMD 0.63; 95% CI 0.34, 0.91; *P* < 0.001), prealbumin (SMD 1.00; 95% CI 0.52, 1.48; *P* < 0.00001), and overall complications (risk ratio 0.73 M-H; fixed; 95% CI 0.58, 0.92; *P* = 0.006).

**Conclusion:**

EN feeding tube support is an essential intervention to elevate patients’ immunity, depress levels of inflammation, and reduce the risk of complications after gastrectomy for gastric cancer. Enteral nutrition improves the innate immune system and nutrition levels but has no marked significance on certain clinical outcomes. Also, EN reduces the duration of hospital stay and cost, significantly.

## Background

Gastric cancer is considered as one of the most common cause of cancer-related mortality globally, with higher incidence in less developed regions across the world [1–3]. A recent retrospective study performed in China showed that gastric cancer is in the top 5 list of most common cancer from 2001 to 2010, although from 2011 to 2015, the incidence of gastric cancer has dropped. This decline in gastric cancer incidence can be attributed to the prevention of gastric cancer through *Helicobacter pylori* eradication [4–7]. Malnutrition is common in gastric malignancies generally due to decreased nutritional intake, which can be aggravated by the side effects observed from cancer therapy and the tendency of cancer to release chemicals containing toxins. These factors are believed to be visible in cancer patients with features of severe loss of body mass, nitrogen imbalance, and fatigue. These characteristic anorexigenic factors require feeding tubes in gastric malignancy resections both in nourished and malnourished patients [8].

Diagnosis of gastric cancer is made when the patient is weak, old, in constant decline of organ function, undernourished, and in a cancerous physique [9, 10]. Despite the agony and stress inflicted, surgery is irrefutably the “gold standard” in the treatment of advanced gastric cancer. Hence, the development of enhanced recovery after surgery (ERAS) has acknowledged minimal invasive procedures as they show similar desired outcomes [11, 12]. Albeit its competency, studies have shown that gastrointestinal cancer patients who underwent surgery also tend to have a concomitant progression of malnutrition, decrease in body weight, increase in hospital stay, and increase incidence of surgical and non-surgical mortality, as well as higher costs of complicated long-term treatment. As such, the prognosis of gastric cancer should involve frequent patient reviews by dieticians [13–15]. Feeding tubes are not widely recommended when oral feeding is feasible, since their sepsis complications can be devastating [8, 16]. Alternative use of EN or PN has helped define the future implementation of feeding in neoplastic patients in the restoration of optimal metabolism and has improved the body’s capability to repair and replace damaged cells to support patients’ innate immunity [9, 17]. Feeding tubes are indicated when energy and nutrient goals cannot be met by an oral mode of nutrition [8, 18].

Nutrition, as a mode of intervention in surgical patients, begun with PN that was used to restore utilized energy and seemed to improve patients’ general outcomes. Its result of stabilizing body weight was impressive. However, analyses indicated that the body weight factor increases extracellular mass but not muscle mass, as thought to be, and it is costly [19]. In addition, unlike EN, PN bypasses the gastrointestinal tract (GIT), which causes effects on the GIT such as decreasing the brush border hydrolase and nutrient transporter activity, increasing permeability, and decreasing microvillus height. These factors caused by PN are believed to result in damaging changes to the physiology of the gut. Parenteral nutrition was then indicated only when EN was less efficient in postoperative rehabilitation. Studies have indicated that EN can also be presented with complications including incidence of pulmonary infections as a result of gastric reflux, anastomotic leakage, diarrhea due to an imbalance in the intestinal flora, irritation caused by chronic micromovements, and foreign body reaction instigating discomfort. Dedes et al. reported case studies with an incidence of bezoar formation when using EN feeding tubes. Other researchers have found out that using EN either perioperatively or postoperatively is beneficial in aspects such as immunity, decline risk of infections, preservation of gut structure and function, prevention of translocation of intestinal bacteria, promotion of normal blood supply to the gut, wound healing early recovery, and decreasing of hospital stay and cost [19–25]. Moreover, studies have demonstrated that the use of supplements added to EN after surgery in nourished patients has no significance when compared to standard EN [17]. Generally, EN is deemed superior to PN, as the former can sustain gut barrier integrity and overall reduction of sepsis complications, as well as decreased mortality incidence and cost. Enteral nutrition benefits on cancer patients’ prognosis are also observed as studies show that patients under EN are also seen to endure more doses of chemotherapy after surgery as compared to PN. Administration of EN utilizes the gut. In contrast, PN provides nutrients directly into the bloodstream, but this can be fatal when severe blood sepsis occurs from catheter-related infections. So, PN is considered only when the gut function is dormant and EN contraindicated [8, 19, 20, 26–28]. Enteral nutrition stimulates gastrointestinal secretions and endogenous hormonal secretions that are important in advancing intestinal adaptation and increase intestinal absorption as compared to oral nutrition and PN. It also accelerates postoperative adaptation to surgical trauma, redeems stomach mucosal functions, and lessens infection complications in comparison to PN [18, 19].

Despite the variations between EN and PN in aspects of clinical outcomes, both Elke et al. and Feng et al. indicated no difference in mortality between the 2 modes of nutrition but a significant reduction of infections in patients under EN. Feng et al. also reported a decrease in organ failure rates in the EN group, whereas Zhao et al. indicated that adding fiber and probiotics in EN could reduce the incidence of diarrhea. A recent randomized control study showed that PN is as safe as EN when delivered according to the recognized practice. However, this result did not indicate that PN is superior to EN [29–31]. Promoting the innate immune system in malignant patients should be crucial since the weakened body’s immunity is certain upon the diagnosis of cancer; an intact immune system should be able to dismantle cancer cells as soon as they appear [32, 33]. Concept modification in the surgical field has instigated a compound of evidence-based medicine to reciprocate the postoperative side effects. Enhanced recovery programs play a crucial role in counteracting intraoperative and postoperative dilemma. Maintenance of body metabolism and conservation of organ functions in response to surgical stress is optimal in the concept of multimodal ERAS, especially with minimally invasive techniques, as the magnitude of inflammation is greater in open surgeries [34]. Compliance to ERAS has been challenging since there have been different opinions regarding different diseases and conditions addressed in the surgical field. The evolution of enhanced recovery after surgery has been acknowledged in several surgical procedures worldwide due to its commitment to positive clinical outcomes.

Post-surgery patients are only allowed to take clear fluids for about 3 days. The 3-day fasting from nutritious contents will further deteriorate energy and immune levels unless feeding tubes are used. This sequence of nutritional practice after surgery is very common in our modern practice. ERAS coincides with the concept of no feeding tubes after surgery, but to achieve the goal of ERAS, a boost in the immune levels and energy levels of the patients is imperative. Despite feeding tubes being contraindicated due to their bitter nature of irritation, risk of infection, and limited mobility in ERAS protocols [35], their importance is being undermined as the agonizing distress caused by malignancies requires a considerable care on the innate defense system. Our primary objective was to find out if there was any importance in feeding tubes corresponding with immune levels, nutrition values, energy levels, and the incidence of postoperative complications in neoplastic gastric resections.

## Methods

### Retrieval strategy

**Fig. 1:**
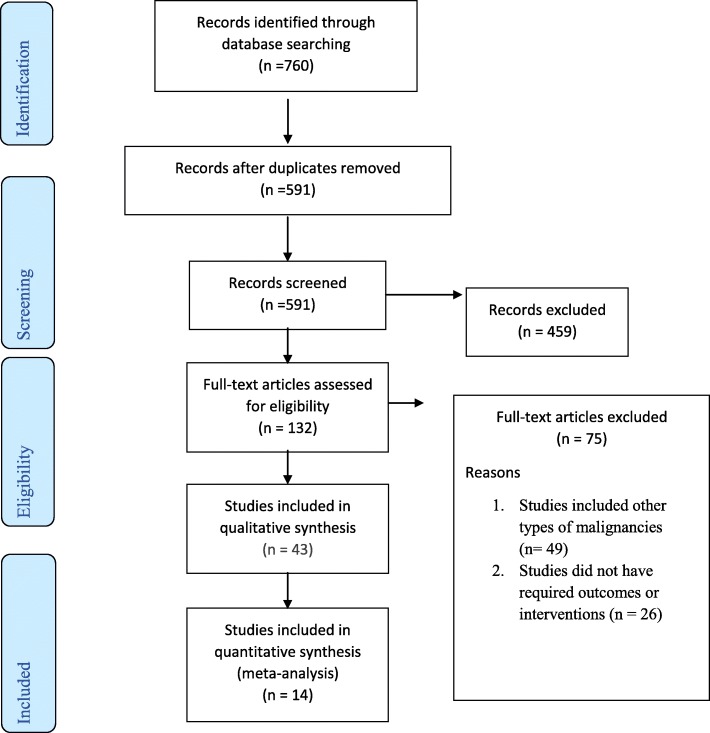
PRISMA flow diagram of literature search and details of selected eligible studies for inclusion in meta-analysis. The PRISMA Statement. PLoS Med 6(6): e1000097. doi:10.1371/journal.pmed1000097

During our search period, qualitative search was not limited to a time frame. However, the quantitative search was from January 2005 to May 20, 2019. PubMed and Cochrane databases were searched for studies involving enteral nutrition (EN) in comparison to parenteral nutrition (PN) in gastric cancer patients undergoing gastrectomy. PubMed search strategy included ((Gastric cancer[MeSH Terms]) AND “enteral nutrition”[MeSH Terms]) AND “parenteral nutrition”[MeSH Terms], ((Gastric cancer[MeSH Terms]) AND “enteral nutrition”[MeSH Terms]) AND “parenteral nutrition”[MeSH Terms] Sort by: Best Match, (((“gastric cancer”[Title/Abstract]) AND “enteral nutrition”[Title/Abstract]) AND “parenteral nutrition”[Title/Abstract]) (enteral nutrition) AND parenteral nutrition, review literature gastrointestinal neoplasms (“enhanced recovery after surgery”[Title/Abstract]) AND “stomach neoplasms”[MeSH Terms]. Search strategy used in Cochrane included ‘gastrectomy and immunity in Trials,’ gastric neoplasms epidemiology in All Text ‘enteral nutrition in Title, Abstract, Keywords and gastric cancer in Title, Abstract, Keywords in Trials,’ ‘enteral feeding and gastric cancer in Title, Abstract, Keywords in Trials.’ PubMed yields 440 selected studies and Cochrane 320. This study was carried out under the PICO system and was reported using the Preferred Reporting Items for Systematic Review and Meta-analysis (PRISMA). The PRISMA flow diagram is illustrated in Fig. 1. Participants (P) included patients with gastric cancer who underwent gastrectomy, intervention (I) included gastric resection with feeding tubes, comparison (C) included EN feeding tubes versus PN, and outcome (O) included immune T cell subsets (including CD3+, CD4+, CD8+, CD4+/CD8+ ratio, NK Cells), biochemical indices (including total protein, albumin, prealbumin, transferrin), overall postoperative complications, hospital stay, and cost which were the endpoints of the present study.

### Inclusion and exclusion criteria

#### Inclusion criteria

Studies were included if participants had resectable gastric cancer and postoperative comparative studies that received nutrition by feeding tubes including nasogastric, orogastric, naso-jejunum, and naso-enteral with parenteral nutrition. Studies were also deemed eligible if patients used feeding tubes only for nutritional purposes, but not other purposes such as decompression. Articles were not limited to the English language.

#### Exclusion criteria

Studies that addressed malignancies other than gastric malignancy and/or resections other than gastric resections and studies that combined the use of EN and PN were excluded. Perioperative use of feeding tubes was ineligible even if they compared EN and PN in gastric cancer.

### Statistical analysis

All analyses were conducted by Review Manager (RevMan) 5.3 and SPSS version 22. Continuous data were expressed as standard mean difference (SMD) or mean difference (MD) with 95% confidence interval (CI), and risk ratio (RR) was used to estimate the dichotomous outcomes. Random effect and fixed effect models were computed under statistical methods of either Mantel-Haenszel (for RR) or inverse variance (for SMD and MD), respectively. Heterogeneity among studies was assessed using the inconsistency statistic (*I*^2^). If *I*^2^ was < 50%, the eligible studies were considered to be homogenous; hence, the fixed effect model was used. In contrast, if *I*^2^ was > 50%, the pooled results were said to be significantly heterogeneous, and the random effect model was used instead. Publication bias was assessed by Begg’s and Egger’s linear regression test (Cochrane handbook version 5.1.0).

## Results

### General characteristics

This meta-analysis included 14 studies comparing EN and PN in gastric cancer resections. There were 10 papers from China, 3 papers from Japan, and 1 paper from Korea: Li et al. [36], Wang et al. [37], Li et al. [38], Chen et al. [1], Liu et al. [39], Li et al. [40], Gao [41], Hongyi et al. [42], Chen [43], Li et al. [44], Nomura et al. [45], Akashi et al. [46], Kamei et al. [47], and Kim et al. [48]*.* Nine papers were randomized with *n* = 1437 participants, and 5 papers were retrospective studies with *n* = 421 participants. The total participants from the randomized studies’ EN feeding tubes group were *n* = 723, and the total participants in the PN group were *n* = 714. The retrospective participants involved EN feeding tubes (*n* = 219) and PN (*n* = 202). The studies (*n* = 14) had participants with a mean age of 60.8321 in the EN group and 60.9786 in the PN group with a total of *n* = 818 females and *n* = 1040 males. Studies that reported on immune indicators included PODs < 7 (*n* = 700) and PODs ≥ 7 (*n* = 995). Studies that included nutrition indices were PODs < 7 (*n* = 1210) and PODs ≥ 7 (*n* = 1334). Finally, postoperative clinical outcomes included a total of *n* = 1657 participants. The reported number of participants in the above three categories was taken from the parameters which produced the highest number of participants. Detailed study characteristics are shown in Table 1.

**Table 1:** Study characteristics

Author (year) [ref]	Country	Diagnosis	Age of patients (years)	Sample size (EN/PN)	Nature of EN	EN initiation time	Gender ratio (women to men)	Mode of enteral feeding tube
Li et al. 2015 [36]	China	Gastric cancer	60.8 ± 5.9 (EN) 56.0 ± 7.6 (PN)	200/200	Standard	Postoperative	96:104 (EN) 88:112 (PN)	Naso-enteral
Li et al. 2015 [40]	China	Gastric cancer	59.2 ± 9.7 (EN) 60.4 ± 9.2 (PN)	150/150	Standard	Postoperative	74:76 (EN) 72:78 (PN)	Naso-enteral
Liu et al. 2012 [39]	China	Advanced gastric cancer	58.4 ± 6.3 (EN) 56.2 ± 6.7 (PN)	24/26	Standard	Postoperative	9:15 (EN) 11:15 (PN)	Naso-enteral
Chen et al. 2014 [1]	China	Gastric cancer	59.4 ± 8.8 (EN) 61.1 ± 7.4 (PN)	37/35	Standard	Postoperative	17:20 (EN) 18:17 (PN)	Naso-jejunal
Li et al. 2015 [38]	China	Gastric cancer	67.7 ± 7.2 (combined EN and PN)	136/136	Standard	Postoperative	92:180 (combined EN and PN)	Naso-enteral
Wang et al. 2018 [37]	China	Gastric cancer	48.07 ± 7.45 (EN) 48.21 ± 6.78 (PN)	66/63	Standard	Postoperative	32:34 (EN) 31:32 (PN)	Jejunum
Gao 2015 [41]	China	Gastric cancer	59.11 ± 8.28 (EN) 59.45 ± 7.14	53/52	Standard	Postoperative	24:29 (EN) 23:29 (PN)	Naso-jejunal
Hongyi et al. 2014 [42]	China	Gastric cancer	65.3 ± 6.7 (EN) 64.9 ± 7.1 (PN)	70/70	Standard	Postoperative	24:46 (EN) 22:48 (PN)	Naso-jejunal
Chen 2014 [43]	China	Gastric cancer	59.5 ± 6.6 (EN) 59.1 ± 5.9 (PN)	50/50	Standard	Postoperative	22:28 (EN) 21:29 (PN)	Nasoenteral/naso-jejunal
Li et al. 2011 [44]	China	Gastric cancer	61.27 ± 10.19 (EN) 60.94 ± 11.26 (PN)	62/54	Standard	Postoperative	14:48 (EN) 14:40 (PN)	Naso-jejunal
Nomura et al. 2015 [45]	Japan	Gastric cancer	66.1 ± 7 (EN) 63.5 ± 12 (PN)	21/22	Standard	Postoperative	10:11 (EN) 6:16 (PN)	Nasogastric
Kim et al. 2012 [48]	Korea	Gastric cancer	Mean age: 60 (EN) 64.5 (PN)	17/16	Standard	Postoperative	5:12 (EN) 3:13 (PN)	Naso-jejunul
Akashi et al. 2012 [46]	Japan	Gastric cancer	65.0 ± 9.5 (EN) 66.7 ± 11.1 (PN)	29/21	Enhanced	Postoperative	5:24 (EN) 6:15 (PN)	Naso-intestinal/jejunal
Kamei et al. 2005 [47]	Japan	Gastric cancer	62 ± 10 (EN) 65 ± 11 (PN)	27/21	Standard	Postoperative	8:19 (EN) 5:16 (PN)	Orogastric

### Analysis of immune indicators

Immune indicators were compared between EN and PN within PODs < 7 or PODs ≥ 7 and beyond. Five studies [36, 39–42] reported on the immune indicators.

#### CD3+ T cells

Levels of CD3+ T Cells were observed among two studies on POD < 7 (*n* = 700) [36, 40] and 3 studies reported on POD ≥ 7 [36, 40, 41]. Heterogeneity among studies was found on both durations, PODs < 7 and PODs ≥ 7. Thence, random effect model was used for the analysis to calculate the effect sizes on PODs < 7 (*χ*^2^ = 101.26 *P* < 0.00001, *I*^2^ = 99%) and PODs ≥ 7 (*χ*^2^ = 67.91, *P* < 0.00001, *I*^2^ = 97%). On PODs ≥ 7, a significant increase in CD3+ T cells in the EN group compared to the PN group was computed (SMD 1.71 (95% CI 0.70, 2.72), *P* = 0.0009), whereas PODs < 7 demonstrated no statistically significant difference in CD3+ T Cells between the two groups (SMD 0.68 (95 % CI − 0.95, 2.30), *P* = 0.41).

**Fig. 2:**
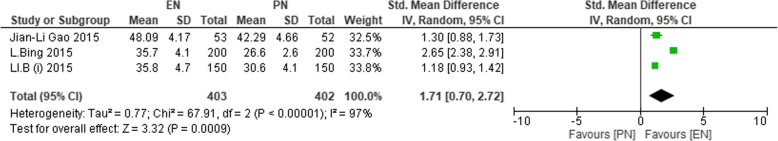
SMD comparing enteral nutrition (EN) and parenteral nutrition (PN) during postoperative days greater than or equal to 7 on CD3+ T cells

Figure 2 shows CD3+ T Cells on PODs ≥ 7.

#### CD4+ T cells

Five studies had eligible data recorded with participants (*n* = 995) on POD ≥ 7 [36, 39–42], 497 made the EN group and 498 made the PN group, while 3 studies reported on POD < 7 [36, 40, 42] with total participants of *n* = 840 having 420 participants in both EN and PN groups. There were some missing data from two studies on postoperative PODs < 7 because some values were recorded on POD 1 while EN began on POD 2 [39]. And data was only recorded 7 days after the initiation of EN and PN [41]. On POD < 7, heterogeneity among studies was observed (*χ*^2^ = 1464.38, df = 2 (*P* < 0.00001); *I*^2^ = 100%), and as the result, random control model was used. This analysis had no significance between the two groups (MD − 1.50; 95% CI − 13.94, 10.94; *P* = 0.81).

On POD ≥ 7, heterogeneity was also observed (*χ*^2^ = 39.16, df = 4 (*P* < 0.00001); *I*^2^ = 90%); thence, random effect model was also selected. CD4+ had significance on POD ≥ 7 (MD 5.84; 95% CI 4.19, 7.50; *P* < 0.00001).

**Fig. 3:**
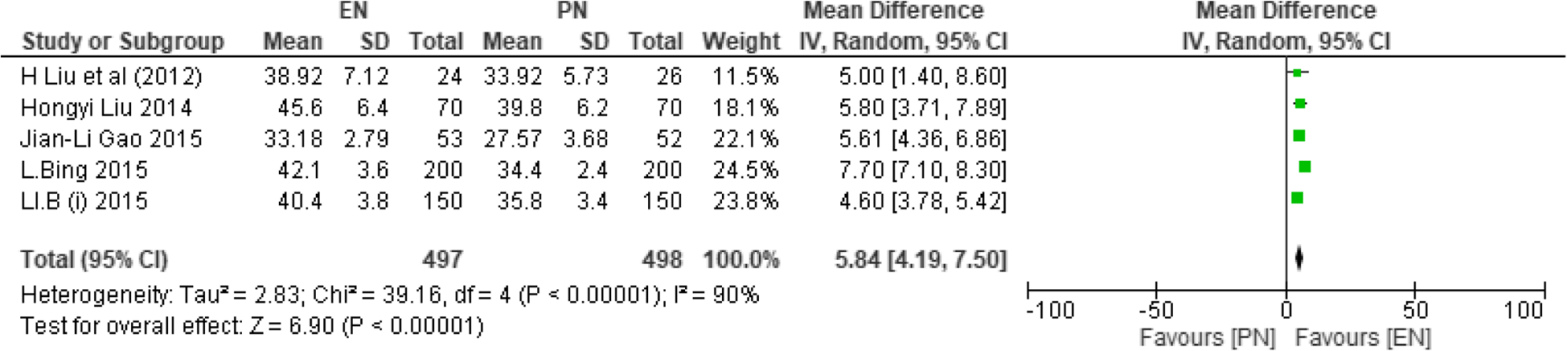
SMD comparing enteral nutrition (EN) and parenteral nutrition (PN) during postoperative days greater than or equal to 7 on CD4 T cells

Figure 3 shows CD4+ T cells on PODs ≥ 7.

#### CD8+ T cells

Two studies reported on POD < 7 [36, 40] with a total number of participants (*n* = 700) having 350 in each group. Two papers [39, 41] could not be recorded since the data provided was on the seventh postoperative day. On PODs ≥ 7 [36, 39–41], *n* = 855 participants were recorded with 427 in EN and 428 in PN, respectively. Heterogeneity among the studies was observed (*χ*^2^ = 123.34, df = 3 (*P* < 0.00001); *I*^2^ = 98%); thus, the random effect model was selected. This was the only immune value with no significance on PODs ≥ 7 (SMD − 0.48; 95% CI − 1.48, 0.51; *P* = 0.34).

**Fig. 4:**
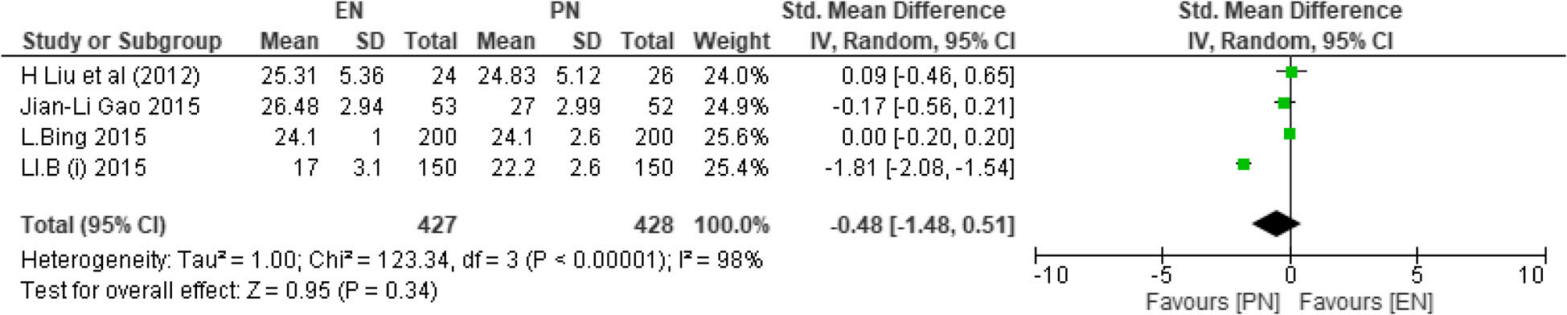
SMD comparing enteral nutrition (EN) and parenteral nutrition (PN) during postoperative days greater than or equal to 7 on CD8+ T cells

Figure 4 depicts CD8+ T cell levels on PODs ≥ 7.

#### The ratio of CD4+/CD8+ T cells

In 2 studies on PODs < 7 [36, 40], homogeneity was evident (*χ*^2^ = 0.15, *P* = 0.70, *I*^2^ = 0%). Thus, the fixed effect model was used to measure the effect size. The pooled estimate showed a significant difference in favor of the EN group (MD 0.22; 95% CI 0.18, 0.25; *P* < 0.00001).

**Fig. 5:**
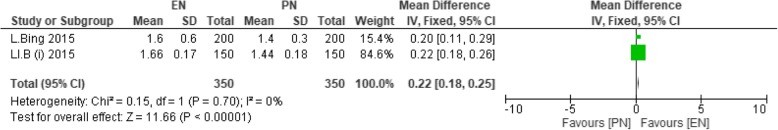
MD comparing enteral nutrition (EN) and parenteral nutrition (PN) during postoperative days less than 7 on the CD4+/CD8+ T cells

Figure 5 illustrates CD4+/CD8+ T cells on PODs < 7.

In PODs ≥ 7, 3 studies were recorded (*n* = 805) [36, 40, 41]. Mean difference (MD) was used as the effect measure. Homogeneity across the studies was observed (*χ*^2^ = 4.76, *P* = 0.09, *I*^2^ = 58%). Hence, the random effect model was used to compute the effect size. Statistical significance was in favor to the EN group, as the ratio of CD4+ and CD8+ T cells increased significantly in the EN group (MD 0.28; 95% CI 0.20, 0.36; *P* < 0.00001).

#### NK cells

On POD < 7, NK cells were reported from 3 studies (*n* = 840), with 420 participants in each EN and PN group [36, 40, 42]. On PODs < 7, an obvious statistical heterogeneity across the two studies was observed (*χ*^2^ = 41.28, *P* < 0.00001, *I*^2^ = 95%). Therefore, the random effect model was selected to estimate the effect size. The pooled result showed no statistically significant difference between the two study groups on PODs < 7 (SMD 0.28; 95% CI − 0.37, 0.94; *P* = 0.40).

Five studies reported on PODs ≥ 7 [36, 39–42], (*n* = 995) with 497 and 498 participants in EN and PN, respectively. Significant heterogeneity among the studies was noticed (*χ*^2^ = 31.48, df = 2 (*P* < 0.00001), I^2^ = 87%). Thus, the random effect model was used to analyze the effect size. There was a significant increase in NK cell count in favor of the EN group (SMD 0.94; 95% CI 0.54, 1.34; *P* < 0.00001).

**Fig. 6:**
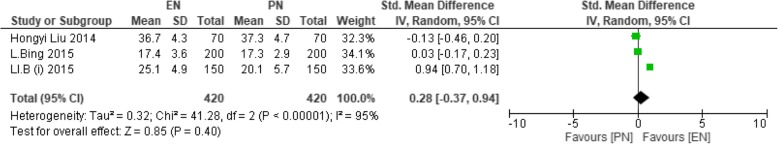
MD comparing enteral nutrition (EN) and parenteral nutrition (PN) during postoperative days greater than or equal to 7 on the NK cells T cells

Figure 6 indicates the level of NK cells on PODs ≥ 7.

### Analysis of nutrition indices

#### Albumin levels

The levels of albumin on POD < 7 were reported in 6 studies [1, 36, 38–40, 44], with (*n* = 1210) 609 participants in EN and 601 in PN groups. Heterogeneity was significant among studies that reported on PODs < 7 (*χ*^2^ = 44.98, *P* < 0.00001, *I*^2^ = 89%), and PODs ≥ 7 was reported from 7 studies [1, 36, 38–40, 43, 44] (*χ*^2^ = 34.29, *P* < 0.00001, *I*^2^ = 83%). Therefore, the random effect model was used to calculate the effect sizes for both periods. On PODs < 7, no significant difference between the two groups was found (SMD 0.09; 95% CI − 0.28, 0.45; *P* = 0.65).

**Fig. 7:**
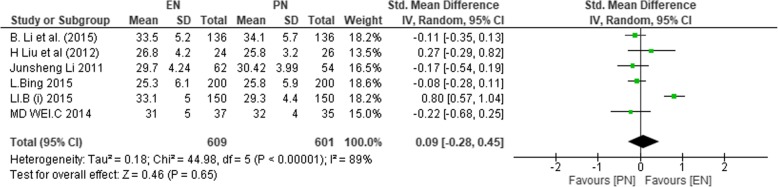
SMD comparing enteral nutrition (EN) and parenteral nutrition (PN) during postoperative days less than 7 on albumin

Figure 7 shows albumin levels on PODs < 7.

A significant difference between the two groups was observed, as albumin levels significantly increased in the EN group on PODs ≥ 7 as compared to the PN group (SMD 0.63; 95% CI 0.34, 0.91; *P* < 0.001). The EN group had significantly increased albumin levels as compared to the PN group.

**Fig. 8:**
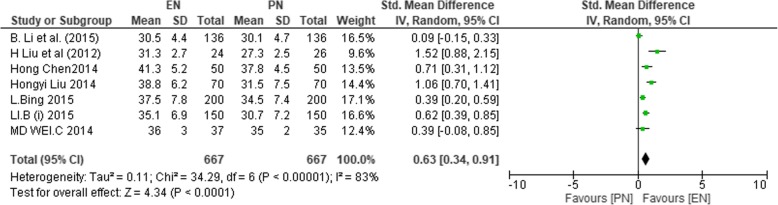
SMD comparing enteral nutrition (EN) and parenteral nutrition (PN) during postoperative days greater than or equal to 7 on albumin levels

Figure 8 shows albumin levels on PODs ≥ 7.

#### Prealbumin levels

Three studies presented data on prealbumin [36, 37, 40]. There were 416 and 413 participants in the EN and PN groups, respectively. Other studies [1, 38, 39] were excluded due to a significant difference in magnitude on the studies. On PODs < 7, heterogeneity among eligible studies was present (*χ*^2^ = 20.07, df = 2 (*P* < 0.0001), *I*^2^ = 90%). Thus, the random effect model was used to estimate the effect size. The pooled result failed to demonstrate any statistically significant difference between the two treatment arms (SMD 0.39; 95% CI − 0.07, 0.85; *P* = 0.10).

In PODs ≥ 7, heterogeneity across studies was present (*χ*^2^ = 20.08, *P* < 0.0001, *I*^2^ = 90%). Thereby, the random effect model was used to estimate the effect size. Comparatively, the EN group had a significant increase in prealbumin levels (SMD 1.00; 95% CI 0.52, 1.48; *P* < 0.00001).

**Fig. 9:**
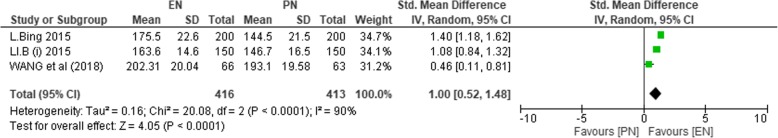
SMD comparing enteral nutrition (EN) and parenteral nutrition (PN) during postoperative days greater than or equal to 7 on prealbumin levels

Figure 9 depicts prealbumin levels on PODs ≥ 7.

#### Transferrin levels

This parameter could only analyze PODs ≥ 7 due to lack of data on PODs < 7. This analysis included 160 and 162 participants in the EN and PN groups, respectively. Data recorded were from two eligible studies [38, 39]. Significant homogeneity was found among the two studies (*χ*^2^ = 0.63, *P* = 0.43, *I*^2^ = 0%). Therefore, the random effect model was used to compute the effect size. Transferrin levels increased on PODs ≥ 7, with a statistically significant difference among the two interventions, in favor of the EN group (SMD 0.68; 95% CI 0.46, 0.91; *P* < 0.00001).

#### Protein levels

Data on protein indices were extracted from 2 studies (*n* = 179) [37, 39] with 90 participants in the EN group and 89 in the PN group. Data from 1 study [1] were excluded due to the difference in magnitude as compared to the other 2 studies. On both PODs ≥ 7 and POD < 7, heterogeneity was evident. Hence, the random effect model was used to calculate the effect sizes for both periods (*χ*^2^ = 5.27, df = 1 (*P* = 0.02), *I*^2^ = 81%, and *χ*^2^ = 60.43, df = 1 (*P* < 0.00001), *I*^2^ = 98%). On PODs < 7, no significant difference between the two treatment arms was found (SMD 2.24; 95% CI − 0.95, 5.43; *P* = 0.17), neither did the result on PODs ≥ 7 show any significant difference between the two groups in terms of total protein levels (SMD 0.62; 95% CI − 0.17, 1.41; *P* = 0.12).

### Analysis of postoperative clinical outcomes

#### Postoperative fever

This analysis included 2 studies (*n* = 700) [36, 40], with 350 participants in each of the treatment groups. Heterogeneity across studies was significant (*χ*^2^ = 53.40, df = 1 (*P* < 0.00001); *I*^2^ = 98%). Therefore, the random effect model was used to estimate the effect size. Patients offered EN had significantly decreased the duration of postoperative fever, as compared to those who received PN (SMD − 1.77; 95% CI − 3.07, − 0.47), *P* = 0.008).

#### Postoperative anal exhaustion time

Four studies (*n* = 1022) reported on anal exhaustion time [36, 38–40], with 512 and 510 participants in the EN and PN groups, respectively. Due to significant heterogeneity among the four studies (*χ*^2^ = 173.19, df = 3 (*P* < 0.00001), *I*^2^ = 98%), the random effect model was employed. A significantly decreased anal exhaustion time in favor to the EN group was observed (SMD − 2.22; 95% CI − 3.47, − 0.97; *P* = 0.0005).

**Fig. 10:**
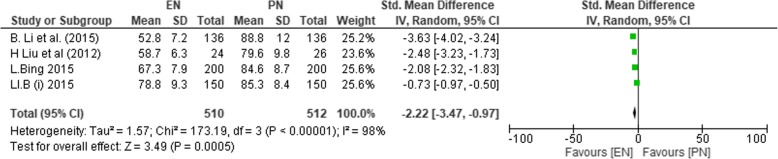
SMD comparing enteral nutrition (EN) and parenteral nutrition (PN) on anal exhaustion

Figure 10 indicates the postoperative anal exhaustion time.

#### Postoperative wound infections

This analysis included 6 studies (*n* = 458) [1, 37, 39, 45, 47]. Heterogeneity was evident among the studies (*χ*^2^ = 6.21, *P* = 0.29, *I*^2^ = 19%). Therefore, the fixed effect model was used to compute the effect size. There was no statistically significant difference in the incidence of wound infections between the two groups (RR 0.65; 95% CI 0.32, 1.31; *P* = 0.23).

**Fig. 11:**
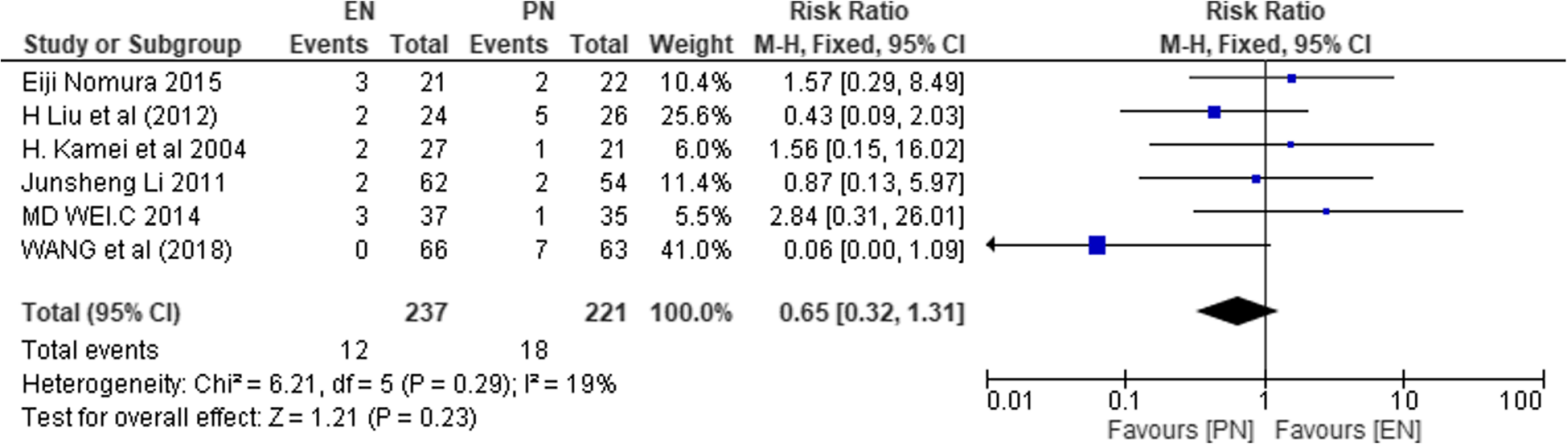
SMD comparing enteral nutrition (EN) and parenteral nutrition (PN) on wound infection

Figure 11 illustrates the result on postoperative wound infections.

#### Pulmonary infections

This involved 4 studies (*n* = 338) [37, 39, 44, 45]. Homogeneity across the studies was significant (*χ*^2^ = 4.88, df = 3 (*P* = 0.18), *I*^2^ = 39%). As such, the fixed effect model was used to compute the effect size. The pooled result demonstrated no significant difference between the two study groups (RR 1.080; 95% CI 0.49, 235; *P* = 0.85).

**Fig. 12:**
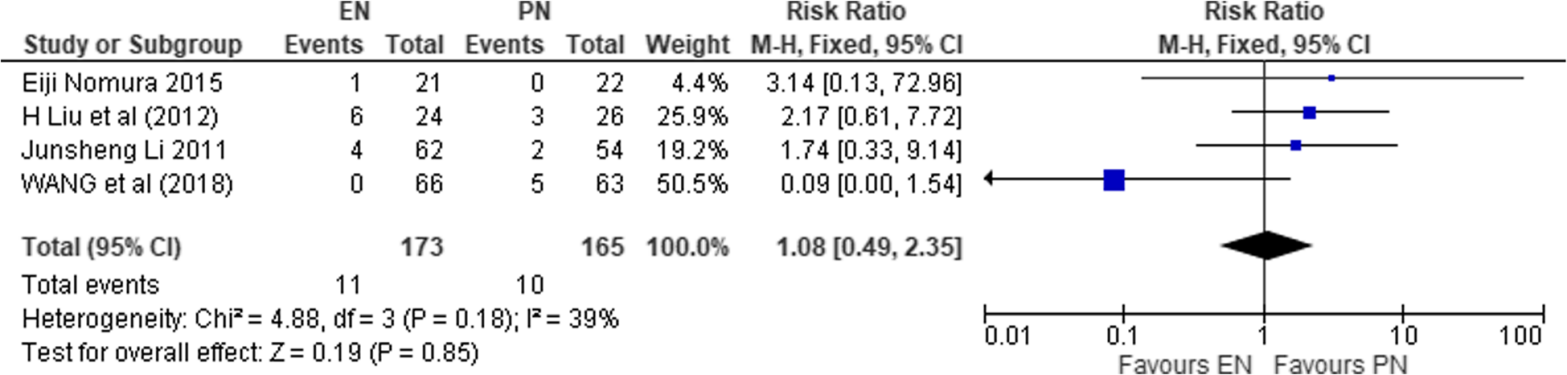
SMD comparing enteral nutrition (EN) and parenteral nutrition (PN) on pulmonary infection

Figure 12 shows the result on pulmonary wound infections.

#### Anastomotic leakage

Six studies (*n* = 510) [1, 37, 39, 43–45] reported on the incidence of anastomotic leakage. No significant heterogeneity among the studies was observed (*χ*^2^ = 9.10, *P* = 0.11, *I*^2^ = 45%). So, the fixed effect model was used to compute the effect size. Results from the pooled estimates indicated no statistically significant difference between the 2 study groups (RR of 1.02; 95% CI 0.99, 1.06; *P* = 0.14).

**Fig. 13:**
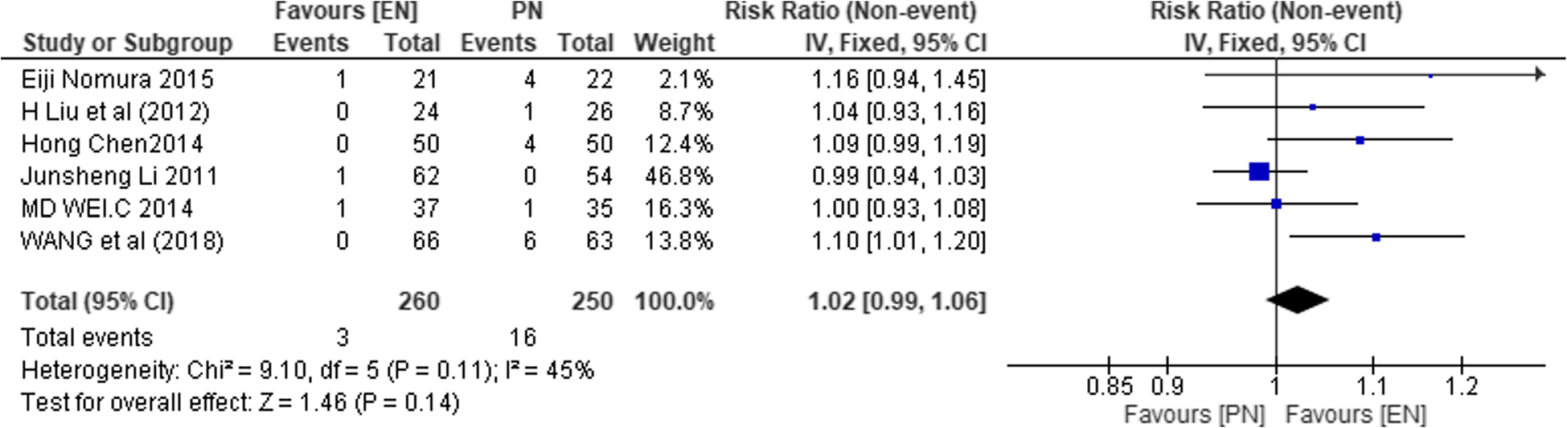
RR comparing enteral nutrition (EN) and parenteral nutrition (PN) on the incidence of anastomotic leakage

The result on anastomotic leakage is shown in Fig. 13.

### Overall incidence of complications

This analysis was estimated with 7 studies (*n* = 848) [38, 40, 43, 45, 46, 48], having 427 and 421 participants in the EN and PN groups, respectively. The studies were homogenous (*χ*^2^ = 5.16, df = 6 (*P* = 0.52), *I*^2^ = 0%); hence, a fixed effect model was selected. The analyzed data showed that the incidence of complications was higher under the PN group whereas EN group had lesser incidence of total complications (risk ratio 0.73 M-H; fixed; 95% CI 0.58, 0.92; *P* = 0.006). This result analyzed that EN participants were less prone to postoperative complications compared to PN.

**Fig. 14:**
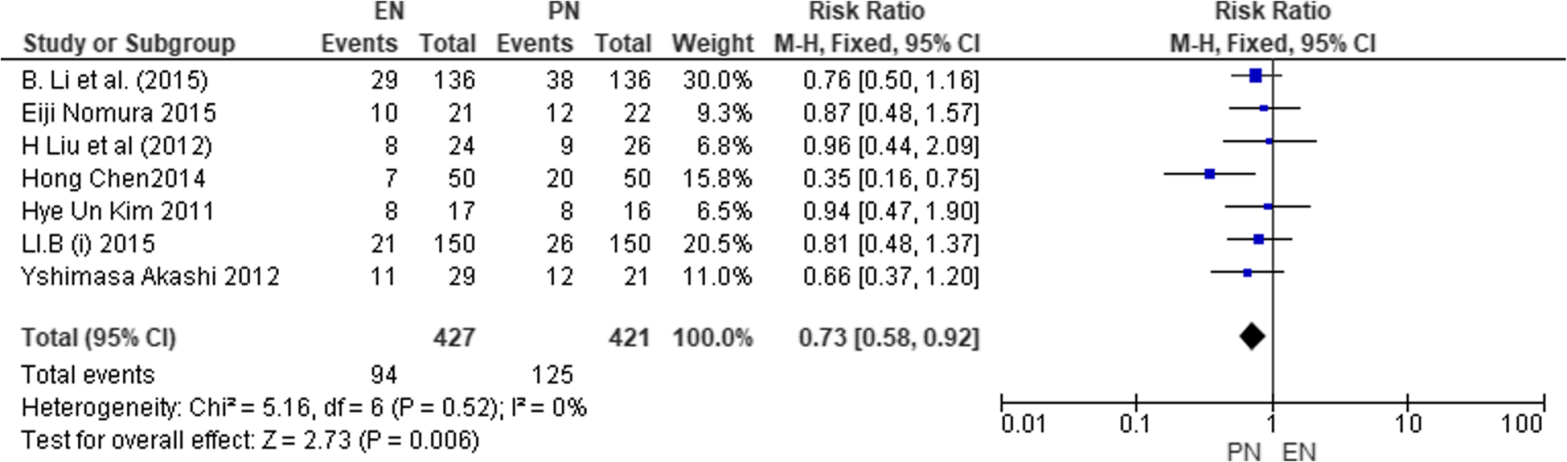
SMD comparing enteral nutrition (EN) and parenteral nutrition (PN) on the incidence of postoperative infections

Figure 14 indicates the result on the overall incidence of complications.

#### Perioperative hospital stay

This analysis included 5 studies (*n* = 571) [1, 37–39, 47], with 290 and 281 patients in the EN and PN groups, respectively. Since the studies were significantly heterogeneous (*χ*^2^ = 9.26, *P* = 0.05, *I*^2^ = 57%), the random effect model was used. Participants in the EN group had significantly shorter perioperative hospital days as compared to the PN group (SMD − 1.08; CI 95% − 1.38, − 0.78; *P* < 0.00001).

**Fig. 15:**
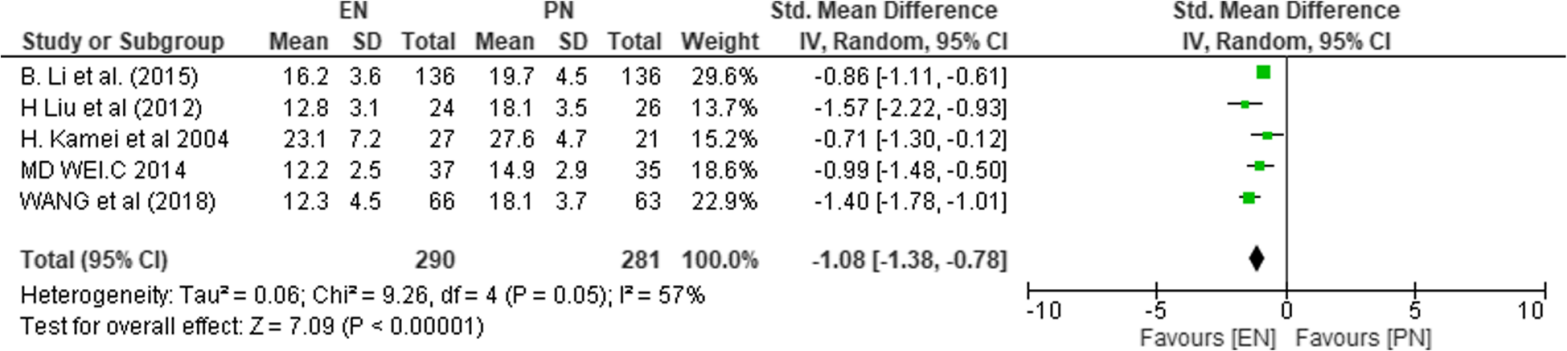
SMD comparing enteral nutrition (EN) and parenteral nutrition (PN) on the total hospital cost

Figure 15 depicts the result on perioperative hospital stay.

#### Total hospital cost

In two studies (*n* = 372) [1, 40], 187 participants received EN, while 185 were offered PN. Homogeneity among the two studies was evident (*χ*^2^ = 0.04, *P* = 0.84, *I*^2^ = 0%). As a result, the fixed effect model was used to compute the effect size. A significant decrease in hospital cost was seen in the EN group as compared to the PN group (SMD − 0.87; 95% CI − 1.08, − 0.65; *P* < 0.00001).

### Assessment of publication bias

**Fig. 16:**
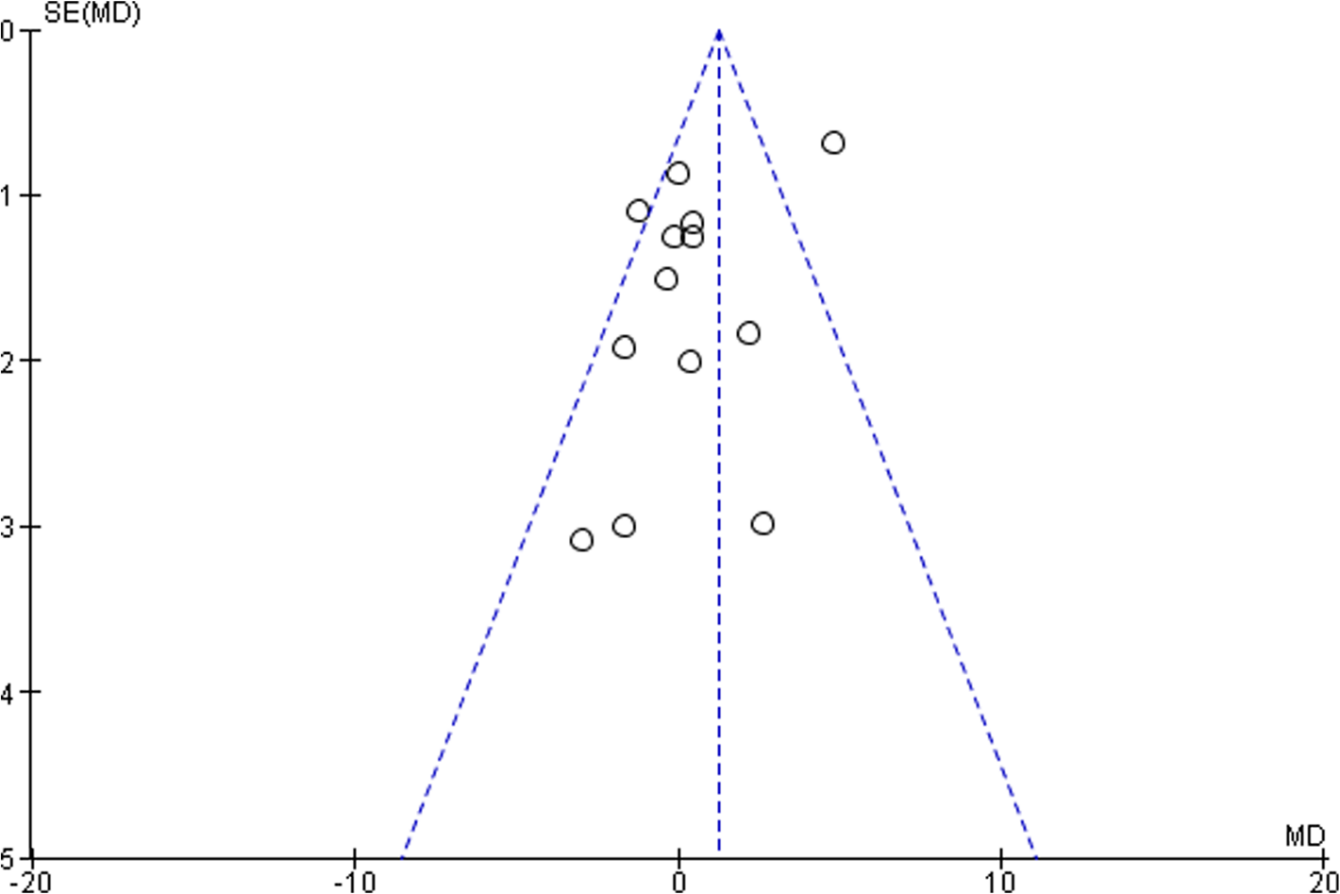
Begg’s and Egger’s test funnel plot for the assessment of bias among studies

The results of Begg’s and Egger’s test indicate publication bias, as shown in Fig. 16.

## Discussion

This study examined innate immune cells such as NK cells, as their capabilities in pro-inflammatory cytokine production and targeted cell lysis that have undergone a malignant transformation or infection are important in the treatment of cancer [49]. Furthermore, levels of CD3+, CD4+, CD8, and CD4+/CD8+ T cells were estimated. Some studies have reported the significance of these cells on the production of interferon (IFN), as well as the phagocytosis of macrophages infected with viral and bacterial antigens [50]. These T Cells were used to investigate the body’s innate response between the two groups. Additionally, the nutrition levels of the patients including albumin, prealbumin, transferrin, and total protein were also used in comparing the two study groups. Both immune and nutritional parameters were estimated as equivalents to equate the possible outcomes of an enhanced recovery including aspects such as shorter hospital periods, since increased postoperative complications are correlated with increased hospital durations and cost.

Our study had 1858 participants, and the final results suggest that postoperative use of feeding tubes have a direct impact on immune T cells, which could have influenced the overall reduction in complication rates that also influences early recovery at a lower cost. Despite some data showing no evidence of statistical significance on postoperative complications such as infections and long-term benefits in the patients’ prognosis (since the duration of intervention was effective for only a short postoperative period and did not include follow-up), clinical parameters that were deemed significant included decreased anal exhaustion time, reduction in postoperative fever time, overall complication rates, and reduction in postoperative hospital length accompanied by lower cost. Time in this study was categorized into two intervals, including postoperative days less than 7 and postoperative days greater than or equal to 7 and not in a form of general postoperative days, since eligible studies differed on the nutrition administration days. We reported an increase in immune levels such as CD3+, CD4+, and CD4+/CD8+ and other indices such as albumin, protein, prealbumin, and transferrin. Results from a recent meta-analysis performed by Cheng et al. indicated no significant difference between EN and EIN in terms of postoperative clinical outcomes such as pulmonary infection (RR = 1.02 95% CI 0.16–6.50, *P* = 0.98), postoperative complications (RR = 0.57, 95% CI 0.18–1.53, *P* = 0.24), and wound infection (RR = 0.67, 95% CI 0.12–3.89, *P* = 0.66) even with additional supplements to EN [51]. Our research demonstrates that feeding tubes have immediate effect on increasing the levels of CD4+/CD8+. Our study data also clarifies the idea that postoperative feeding tubes facilitate shorter durations of hospital stay due to reduced incidence of complications. Despite the paucity of significant differences between EN and PN on infections observed in our current study, postoperative fever time and anal exhaustion periods lasted longer in the PN group. Reduction in postoperative fever time was due to a lower incidence of infections or a boosted immune system in the EN group. A previous meta-analysis performed by Chow et al. showed a significant difference in the decrease in the incidence of infection in the EN group, as compared to the PN group (RR 1.09, 95% CI 1.01 to 1.18, *P* = 0.03) [52]. Postoperative oral feeding is the mainstay intervention whenever possible. However, patients with cancer tend to have a reduction in immune surveillance, which needs supplementary support such as feeding tubes to influence early recovery [53]. The ultimate goal of promoting the patients’ recovery should be maintaining the optimal body capacity using protocols based on evidence, which are simpler for the practitioner and the patient to comply with, and at the same time, they should have positive results in favor of the patients’ general outcome. ERAS enforces carbohydrate loading and deprecates the routine use of feeding tubes. But studies show that diets with high carbohydrates are likely to pull water into the lumen of the gastrointestinal tract due to the high osmotic load and the leaky epithelium of the jejunum, thereby precipitating net fluid and electrolyte, and do not improve muscle strength [18, 54]. Evidence-based ERAS society researchers also show that carbohydrate loading prior to surgery has more advantages than the disadvantages. Preoperative carbohydrate loading improves perioperative insulin sensitivity, helps maintain body mass, improves preoperative well-being, and should be used routinely as recommended by the ERAS society [55, 56]. Adjustment in the current ERAS protocols to suit patients’ capabilities and expected outcome due to the type of disease and treatment administered should be made easier to comply with. We emphasize on adding enteral feeding tubes to ERAS protocols for the treatment of patients with gastric malignancy after gastrectomy, since the current neglect of feeding tubes is not manageable, accordingly. This is the first study, to the best of our knowledge, that emphasizes feeding tubes in ERAS patients with gastric malignancies undergoing gastrectomy.

Notwithstanding the important observations made from our present study, the results could have been influenced by the geographical location of the study participants (i.e., Asia) due to limited access to data, the different modes of surgical resections, the cancerous state of individual patients, the duration of feeding tube application, the preoperative complications, and the overall sample size. None of the patients from our study groups were from the ERAS pathways due to insufficient data from our sources; therefore, we used the normal doctrine of surgical patients to illuminate the significance of postoperative feeding tubes in gastric cancer.

## Conclusions

Although our current study is not designed to answer the questions regarding the postoperative use of feeding tubes in ERAS pathways due to the nature of our study design, we show some thought-provoking findings on EN feeding tube in gastric cancer patients. This meta-analysis investigated the immunity and nutritional importance on the patients’ clinical outcomes. Our participants were not under ERAS pathways but rather the normal perioperative pathways. The results indicated an increase in postsurgical immune levels, increase in nutrition levels, lower incidence of overall complications, shorter hospital durations, and low cost. Conversely, there are no differences in pulmonary infections, wound infections, and anastomotic leakage in the group that administered EN via postoperative feeding tubes. Furthermore, the use of feeding tubes was considered to be possible after gastric cancer resections. Further studies comparing protocols of ERAS with ERAS-based participants using feeding tubes and non-feeding tubes under the current ERAS protocols should be performed.

### Future directions

Despite low compliance to ERAS, it is seen to be growing since its protocols are evidence-based. From simple surgical procedures to more complicated procedures, surgeons, anesthetist, patients, and all others involved have received benefits such as uncomplicated surgeries, early recovery, shorter hospital stay, and good quality care on low cost from ERAS. Unfortunately, most surgeons generally focus on surgical technique alone, disregarding the perioperative management of patients altogether [57]. Development of ERAS should now focus on different surgical departments and on different diagnoses. Studies should now consider the individual perioperative requirements in ERAS protocols including enhancing patients’ metabolic capabilities for future treatment plan such as palliative therapy in critically ill patients. Flexibilty of ERAS protocols should be based on the individual patients prognostic requirements.
